# The Surgical Treatment of Amenorrhœa

**Published:** 1864-01

**Authors:** Horatio R. Storer

**Affiliations:** Boston, Surgeon to the Pleasant Street Hospital for Women


### Selections.

### THE SURGICAL TREATMENT OF AMENORRHŒA.

By HORATIO R. STORER, M.D., of Boston, Surgeon to the Pleasant Street Hospital for Women.

Read by invitation before the Norfolk District Medical Society of Massachusetts. 1863.

The medical treatment of amenorrhœa has been fully discussed by a host of writers upon the subject; among the last of them, and very understandingly, by Dr. Wm. E. Bowman, of Montreal, in the *Canada Lancet*, for June, 1863. Inasmuch, however, as there are certain cases of the affection which, in the most skilful hands, resist all of the therapeutic measures instanced, either in our usual text-books, or in the valuable paper alluded to, it may be of practical advantage to take up the subject just where it is there left, namely, in its relations to obstetric surgery.

Cases of amenorrhœa, while among the most frequent of those applying for aid, and, from their very diversity of origin, among the most interesting, have often been found, in practice, the most tedious and discouraging. Certain possibilities also of mistaking their real character, and of unintentionally, and so far innocently, committing malpractice, and thereby of adding confirmation to an opinion already but too prevalent, that the profession directly, or by implication, sanction the induction of criminal abortion, render them among the most important to which our attention is called.

There are two dogmas concerning them, however, that, with all respect for the large and influential class of practitioners by whom they are held, should not be accepted. They are the following, and I prefer to state them as expressed by writers of authority:—

"It is easier," says Churchill in his last edition, "to manage almost any of the other curable diseases to which females are obnoxious."*

"All attempts," remarks Condie, "to bring on the menstrual flux by directly irritating the uterus, whether by the introduction of bougies or not, are unjustifiable; we can conceive of no case in which they would be calculated to do good; they cannot fail, in many cases, to be positively injurious."†

* Diseases of Woman, p. 152.

† Editorial note to Amer. ed. of Churchill, p. 149.

In practice, such statements are daily disproved.

As a general rule, it will be found, recollecting the possibility of an unusually early occurrence of the menstrual climacteric or normal cessation, that amenorrhœa which has resisted all the therapeutic measures usually resorted to, depends, in the absence of pregnacy, upon one or other of the following causes:—

1. That the uterus is absent, or
2. Imperfectly developed;
3. That it has undergone too completely the process of involution after normal labor or an abortion;
4. That the nisus uterinus of menstruation or nervous excitability of the uterine mucous membrane is in abeyance, even though the ovarian excitability, and even the performance of their peculiar function, may be normal and complete;
5. That the cervical canal or its inner orifice is partially contracted, organically, or by tonic or clonic spasm;
6. That there is complete occulsion, congenital or acquired, of either the uterus, vagina, or external organs;
7. That there exists metritis, acute or chronic, or some other general affection of the uterine mucous membrane, or a chronic retention within its cavity, as of fœtal debris;
8. That there is lesion, usually inflammatory, of one or both ovaries; producing its effect, perhaps, by derivation, or prevention of the usual and normal sanguineous determination;
9. That there is present either general toxæmia, some distant organic disease, by its local congestions preventing the usual afflux of blood to the uterine system, or some distant functional disturbance giving rise to a drain upon the general system, whether hemorrhagic, diaphoretic, leucorrhœal, diabetic, or lacteal.

Any of these varieties, though more frequently the latter, may be attended by vicarious menstruation from distant surfaces, causing often the gravest errors respecting the true character of the disease; a fact that explains many of the cures of so-called phthisis, chronic dysentery, &c., by irregular practice.

The several classes instanced are all more or less commonly met with, and are all amenable to treatment. From this statement I do not even except certain instances of the first class, those where the uterus is entirely wanting, which require, as will be seen, even more judgment and skill than any of the others.

Depending upon such varied causes, it is evident that the utmost care is necessary in the differential diagnosis of any given case; and though I do not now propose to discuss the respective characteristics of either or any of the different varieties, there are yet certain surgical considerations bearing upon this point, of such importance as here to demand mention.

First and foremost; a thorough physical examination of the patient, in cases of amenorrhœa that have resisted all the resources of general treatment, becomes absolutely necessary. As a rule, unmarried women should never in any case be subjected to such examination, unless the existence of organic disease or displacement is undoubted or reasonably suspected, or the woman has previously borne children, until all ordinary strictly medical measures have been brought to bear. On the other hand, married women, presenting any of the symptoms usually diagnostic of uterine or ovarian disturbance, should never be prescribed for until a proper physical examination shall have been effected.

2d. The examination when made, if in the case of an unmarried woman, should generally be aided by an anæsthetic, alike to prevent mental and physical suffering, and by complete relaxation of all pelvic muscular action to facilitate the diagnosis. The usual plea for refusing an anæsthetic at this time, that it is well for the patient to be able to realize her pain, I consider of no weight whatever; so deceptive are the reflex sensations produced by local pressure within the cavity of the pelvis, that it is of far greater advantage to have them altogether absent.

3d. Except in the instance of atresia vaginæ, or some other local lesion absolutely demanding direct vision, the examination, especially in the case of an unmarried woman, should be only by the touch and that longer finger, the uterine sound. The obstetrician should always carry an eye at the end of his forefinger, and it is just as easy to educate the touch to this, as for the blind to their alphabet. Were the use of the speculum confined to the cases where alone it is legitimately required, namely, the comparatively few instances actually demanding local stimulation or cautery, there would be far less quackery in obstetric practice, and far more general appreciation of the real frequency and importance of uterine displacements.

4th. In using the uterine sound, which is here indispensible in many cases to a correct diagnosis, the possibility of pregnancy must be borne in mind, even though the patient be unmarried, no matter how respectable her position, correct her general history, extensive her hymen, or of what standing the absence of her menses; for in default of this precaution many an unexpected abortion has been induced.

This premised, I proceed directly to the surgical treatment of amenorrhœa.

In the first place, the uterus is sometimes absent, and I have said that where this has been diagnosed, there are required extreme judgment and skill. It is often much more difficult to withhold the hand than to attempt aid, but it is evident that in many cases all assistance must be absolutely impossible. There are others, however, where the ovaries exist, and are perhaps well developed, and where there is an evident ovarian molimen. Here the question of attempting treatment will depend upon the theory held by the attandant, as to the essential nature of menstruation, and upon the condition of the patient during these monthly attempts at effective discharge. If the normal catamenia, which must be allowed to be strictly uterine or almost entirely so in their actual origin, are viewed as a mere incident or effect of the irritation or irritability consequent upon ovarian evolution, or as a mere independent excretion for the purpose of conveying from the uterus the unimpregnated ovum, it would hardly seem necessary ever to endeavor to assist nature in the cases to which we are now referring. But if, on the other hand, we grant that this discharge, while partly and by reflex action the effect of the ovarian irritability just alluded to, may also serve as a relief by crisis to any local congestion or disturbance dependent upon the same reflex action, and so evident in almost all cases of disordered menstruation,* and that the uterus besides, as has been well shown by Andral and Gavarret, is intended to act as an accessory respiratory organ,† then it may be thought judicious practice to endeavor to avert such congestion or disturbance by local depletions, dry cupping, &c., to the sacrum, abdomen, or region of the perineum. This treatment is undoubtedly indicated in plethoric patients, where the uterus being absent there is yet a molimen, and I am inclined to think it of importance in others, even where general congestion may not be present.

Secondly, the uterus being present, but as yet undeveloped. Here let me allude to the fact that conceptions may take place even though menstruation may never have occurred, and though

* My eighth class, where suppression of the menses accompanies ovaritis, though at first sight contradictory of the above hypothesis, really goes to confirm it; actual inflammation being a very different thing from a normal and strictly physiological congestion, and productive of wholly different effect.

† The importance, in this connection, of the above views I have elsewhere more than once insisted upon. They tend to explain the otherwise inexplicable fact of the immunity of chloroform from ill result during labor, the altered ratio of progress of phthisis during pregnancy, and other thoracic problems, and they account for the excellent results in the comparatively not uncommon cases of so called uterine asthma, which are almost always accompanied by amenorrhœa, obtained from the use of intra-uterine pessaries.

the uterus may be far smaller than usual, provided only that its canal and those of the Fallopian tubes are sufficiently developed to admit the passage of either ovum or seminal fluid. I speak decidedly on this point, for I am satisfied that conception has taken place not merely in the comparatively frequent instance of periodical leucorrhœa or colorless menstrual discharge, but even when no critical discharge whatever has been recognized.

The indication in these cases is on the one hand to stimulate the lining membrane of the uterus to its proper excretory action, and on the other to excite structural growth and increase in the several component tissues of that organ. Many of the measures by which this has been attempted have utterly failed; this is true of galvanism, however and wherever applied externally to the uterus, of all applications to the os or vaginal wall, of all attempts to produce reflex or sympathetic action by irritation of the rectum, with aloes, &c., and of the exhibition or ergot, here based upon an erroneous theory—for even if it were possible to excite uterine contraction in such cases by ergot, or I might almost add in any case unconnected with pregnancy at nearly the full period, it is not by such transient muscular efforts that we are to insure structural growth. In these cases it becomes necessary to invade the uterine cavity, and to have recourse to the means so ingeniously suggested to the profession, above all other men, by Simpson, of Edinburgh.

By the intra-uterine air-pump, the first of the two indications insisted upon may be fulfilled; I have more than once succeeded in thus inducing and keeping up the normal menstrual discharge when it had never before appeared. But this method has certain drawbacks; the instrument cannot be introduced unless there exists quite decided patency of the os, cervical canal and uterine cavity—it almost necessitates laceration of the mucous membrane, which under forcible suction must enter the open fenestræ, or even minute perforations, of the instrument, to which also it clings when this is withdrawn.

To the intra-uterine stem pessary, however, no such objections apply, and after an almost constant experience of its use for now nearly nine years, I do not hesitate to decidedly recommend it. Against this instrument very many objections have been urged; all of them, however, unfounded, provided certain precautions are taken.

The intra-uterine pessary, as I have elsewhere insisted,*

* Preface to Simpson's Obstetric Works, Amer. ed., pp. 16, 18.

For the purpose, as here, of producing intra-uterine action alone and not of remedying a displacement, it is not at all requisite that the instrument should should perfectly fit the patient; that is, the diameter of its stem should be of such size as readily to enter both the outer and inner openings of the cervix, and its length such as not quite to touch or bear upon the fundus uteri, which can easily be decided by the previous careful use of the uterine sound. If these precautions are attended to, little fear of inducing undue irritation need be entertained.

For the treatment of the cases now under our consideration, and for the purpose of producing an additional therapeutic effect by decided galvanic action, the stem should be formed of copper and zinc, the strips of these metals being generally placed end to end; but perhaps a still better effect would be produced if they were soldered side by side. The amount of action produced in this way is shown by the fact that, upon withdrawing the instrument from the uterine cavity, while the copper portion remains almost entirely free from deposit, that of zinc is found incrusted with a thick layer of foreign matter, which upon chemical analysis resolves itself into the metallic salt usual under similar circumstances.

The effect of the galvanic intra-uterine pessary upon amenorrhœa dependent upon deficient development of the uterus, is of a threefold character; for while the mucous membrane is stimulated to its functional action, a profound impression is produced upon the deeper structures of the uterus both by the direct galvanic influence and by the action of the contained pessary as a foreign body—exciting persistent or at times interrupted muscular action for its expulsion, and thereby a general hypertrophy and increase of growth, as in the case of concealed polypi, etc.

In the third, fourth, and fifth classes of absence of the menstrual discharge that I have enumerated, the same treatment by the galvanic intra-uterine retained bougie is also indicated, and in each it is productive, in a large proportion of cases, of very decided effect. In the third variety the process of atrophy which obtains to a certain extent after ordinary parturition, is carried to an abnormal excess. The cavity of the uterus, instead of its usual size of two and a half inches, measures but an inch and a be provided with the vaginal stem and external clasp; indeed these additions are now seldom required even for preventing displacements, except in extreme and obstinate cases. I am now constantly treating the various versions and flexions of the uterus by Hodge's excellent levers, against which I was formerly prejudiced, but now cannot laud too highly. Should, however, the double instrument of Simpson be found indispensable, I must urge the modification of it proposed by myself in 1856, (*Boston Med. & Surg. Journal, Nov.* 1856, p. 288,) by which a perfect fit to the patient may be insured.

half, an inch, or even less, and with this uterine effacement there often occurs also a cessation more or less complete of the performance of the menstrual function; the case as regards treatment being therefore rendered entirely identical with the class we have just been considering, those, namely, in which the organ has never been fully developed at all.

In some cases, again, there is apparently not the slightest organic deviation present, the ovaries seem to act in their normal manner, and yet the uterus does not respond by its usual flux. There is only wanting, as has been well suggested, a slight initiatory influence, like a touch to the pendulum, to be followed by regularity of menstrual recurrence and discharge.

In the fifth class of our series, which if but partial are generally attended by dysmenorrhœa also, there is required, in addition to mere stimulus and excitement, a certain amount of local dilatation. For this, the instrument already described may of itself at times suffice. Usually, however, I have found further measures required, relying sometimes upon the successive introduction of a graduated series of metallic bougies, and at others upon the use of expansible tents. Eight years ago, I referred to the advantages and disadvantages of sponge for this purpose,* and have found in practice the suggestion I then made, of tents self-lubricating from their own intrinsic mucilage while expanding,† to answer various important indications. As a general rule, I do not favor lateral incision of the cervix, because seldom necessary; but this treatment sometimes becomes indispensable in cases of amenorrhœa dependent upon the cause now described.

* *Boston Med. & Surg. Journal, Nov.* 1855; Simpson's Obst. Works, preface to Amer. ed., p. 16, *Sept.* 1855; this Journal, *Jan.* 1859, p. 57.

† *Association Medical Journal*, London, *May*, 1855, p. 446; *Glasgow Med. Journal, April*, 1856, p. 116.

In practice I have thus far found slippery-elm bark to afford the best material for the special indication above instanced. The sea-tangle (*Laminaria digitata*), a variety of the so-called "devil's apron," or giant rock-weed, has been suggested by Dr. Sloan, of Ayrshire, in the *Glasgow Medical Journal* for *Oct.* 1862, and since referred to by several continental writers. It is acknowledged, however, to enlarge unequally in consequence of its cellular structure, and is liable to "a bulbous expansion forming behind the stricture," as well as to "a marked increase in its length;" both of them decided disadvantages in producing dilatation of the uterus. In this connection I must acknowledge my obligation to Dr. Ephraim Cutter, of Woburn, for a communication from M. Bureau-Riofrey, of Paris, written at the request of Nelaton, containing little, however, in addition to what had already been presented by Dr. Sloan. I am at present investigating the subject, and may therefore yet find occasion to modify my unfavorable opinion. It is possible that the *L. saccharina* of our coast may prove as worthy experiment as the *digitata*, but our most common species, the *longicruris*, I do not hesitate to condemn; its hollow stem

In introducing the several instruments, to which I have alluded, within the os and canal of the cervex, certain precautions are all important. I have already spoken of the possibility of pregnancy, and of the risk in such event of unintentionally inducing abortion. The remark applied with equal force to the sound and intra-uterine bougie, whether these be applied for stimulation, dilatation, or the reduction of displacement. I am well aware of the tolerance and impunity with which at times the pregnant uterus bears such entrance, and have myself had several instances of this brought under my notice; but on the other hand, I have known, from the use of these instruments, more than one direct occurrence of the accident against which I would now guard the profession. I have already more than once referred to this matter in these pages, and have dwelt upon it at some length in my report upon the subject* rendered to the American Medical Association, in 1859; and, from much more extended experience of the true frequency of criminal abortion, I merely reiterate the opinions then expressed; their importance is rapidly becoming acknowledged.

The necessity of extreme caution in deciding upon the existence or not of pregnancy cannot be over-estimated. I speak with the more earnestness in this matter, because it is a point to which I have long given special attention. In a somewhat elaborate discussion, some years since, of the actual and relative value of the several signs of pregnancy usually recognized,† I was compelled to assert that the fœtal pulse is the only one upon which any certain reliance can be placed. This was at variance with the opinion then entertained by my friend, the late Dr. Montgomery, of Dublin, certainly the most eminent authority collapses upon drying, and renders futile any attempt at its preparation or use. Careful examination of many other of our rock-weeds has as yet not furnished me any stems of sufficient size and tenacity for the purpose, the nearest approach to it having been in an unusually large and well-developed specimen of *Fucus vesiculosus*. It is to be regretted that there does not yet exist, as I am informed by our justly celebrated algologist, Dr. Durkee, any collection of the giant sea and rock-weeds of this vicinity. Prof. Agassiz writes me that those formerly collected by himself have been sent to his brother-in-law, Prof. Braun, Director of the Botanical Garden in Berlin, and their is no series of the character desired in the magnificent herbarium of Prof. Asa Gray, as I learn from that gentleman. A very few moments' inspection of such would at once decide as to what species alone our expectations could rationally be based upon.

* Transactions of the Amer. Med. Association, vol. xii, 1859, p. 75; *North Am. Med.-Chir. Review, Jan.* to *Nov.* 1859; Treatise on Criminal Abortion in America, Philadelphia, 1859, p. 70.

† Review of Montgomery's Signs of Pregnancy, *North Am. Med.-Chir. Rev., March*, 1857, p. 249.

upon this subject; shortly previous to his death, however, Dr. Montgomery wrote me that he both accepted and indorsed the limitation I had made.

Pregnancy being assuredly absent, it is only requisite to bear in mind the fact that as in other strictures, of the male urethra, for instance, spasmodic action often suddenly ceases upon long-continued gentle pressure, and allows the entrance of the instrument; and that in the frequent instance of complication with uterine displacement, it is at times necessary slightly to push up the fundus uteri, and so straighten that organ before such entrance can be effected. The cases where, the uterus and ovaries being perfectly normal, there yet exists obstruction or occlusion of the Fallopian tubes, from the extension of general peritonitic inflammation or otherwise, are still beyond all operative aid, despite Tyler Smith's ingenious but impracticable proposal of tubal catheterization. In such instances, a menstrual discharge may or may not regularly take place; if both tubes, however, are closed, the escape of ova or passage of semen, and consequent conception, are manifestly impossible. In these cases the possibility of peri-uterine hæmatocele, from ovarian hemorrhage attending an attempt at ovulation, must be borne in mind.

There remain but three more classes of amenorrhœa remediable by the surgeon. Of these, one, the sixth in our enumeration, where there is complete occlusion, congenital or acquired, of the generative canal, has been amply treated of by many surgical and obstetric writers. One single point regarding it has, however, been almost universally lost sight of, the liability, namely, in these cases, after so remarkably simple an operation, to a fatal result; yet the explanation of this is very simple, and its indication in practice equally plain.

A small incision, which is generally made through fear of occasioning collapse, should the uterus be suddenly emptied, is almost sure, in consequence of the thick and inspissated condition of the fluid usual in retained menses, to occasion powerful uterine contractions after the flow has once begun; just as these take place after labor, from the presence of clots, shreds of membrane, etc., foreign to the uterine cavity. During these contractions, the natural outlet being still impeded, there is no doubt that at times a portion of the retained fluid is forced backward through the Fallopian tubes into the cavity of the abdomen, giving rise to fatal peritonitis. By a free incision of the obstruction, therefore, whenever existing, the uterus should be emptied as rapidly and as thoroughly as possible, even to the extent of completely rinsing its cavity by gentle injections of lukewarm water or soapsuds, and subsequent compression through the abdominal walls, as in a case in which, nearly ten years ago, I assisted my friend, Dr. Malcolm, of Edinburgh.

Previous to this operation, and I am daily more and more inclined to extend the precaution to all that involve the pelvic viscera, it is well to have recourse to the preparatory treatment recommended by Clay, of Manchester, in cases of ovariotomy, giving doses of ox-gall for several days. I am also in the habit, from the analogies obtaining alike in origin, progress, and result, between erysipelas, puerperal and surgical fever, and peritonitis, of depending somewhat upon the preparatory administration of muriate of iron.

The next cause that awaits us is where there exist certain general organic lesions of the uterus itself, its walls or their lining membrane. These affections as causing amenorrhœa are few, hyperæmia of the mucous membrane and menorrhagia being here the rule, and can only be ascertained under dilatation by expansible tents, which may unexpectedly reveal, as it has not unfrequently done, the retained remains of some long past or forgotten conception. It is in the former of these cases, especially where following an attack of metritis, that we find the best results from a direct application to the lining membrane of the uterine cavity, either of nitrate of silver and other stimulants in substance, or in solution, by a sponge. This of course must be done by the aid of instruments specially constructed for the purpose;* and I would suggest that from galvanism thus locally applied by wired sponge through a long and curved canula, as has lately been done in the case of the female bladder,

* I have long been in the habit of using for this purpose an instrument similar to that proposed by Lallemand for the male urethra, but have always been dissatisfied with its size, its complexity, and in recommending it to my friends, its comparatively great cost; the lower portion both of the sheath and the contained stem being necessarily of platinum. Of late, however, I have used an implement of much more simple construction, for the suggestion of which I am indebted to my friend, Dr. Mack, of St. Catharine's, C.W., who seems to have been the first to apply it in practice, although a form closely similar has lately been proposed by Dr. Lente, of New York, (*Amer. Med. Times*, *Sept.* 26, 1863.) Dr. Mack uses a bullet-probe, coated at its tip with a caustic bead, obtained by dipping the slightly heated point of the probe in fused nitrate of silver. To render the instrument more convenient for constant use, I have supplanted the pincers and slide in the usual closed and jointed caustic holder for the pocket, by a stout platinum wire, probe-pointed, which can be easily kept armed with the nitrate, and will be found upon trial to answer a most admirable purpose; the probe may here be made movable, and inserted within the grasp of the stationary forceps when required; at other times being kept in the hollow space of the handle. The instrument thus constructed answers a double purpose, and Messrs. Codman & Shurtleff, instrument makers, of Boston, are now prepared to supply it to the profession.

a much better effect would be obtained than from any other mode in which it has been applied for amenorrhœa. I would decidedly condemn the use of every kind of intra-uterine injection, for whatever purpose indicated, unless, as I have said, to cleanse the cavity after the operation for retained menses; since they are much less easily controlled in action and, for other reasons, are attended with infinitely more hazard.

The last of the divisions described, those depending upon ovarian inflammation, as in many cases of so-called spurious pregnancy, or upon general organic or functional disturbance, would at first sight seem evidently within the domain of strictly medical treatment. On the contrary, it is by a direct appeal to the uterus itself by one or other of the methods to which we have so often alluded, that we must sometimes combat the existing lesion or functional abberration; in many cases of incipient phthisis, for instance, where there has been no vicarious hemorrhagic discharge, and consequently no error of diagnosis, the disease has thus been warded off, or if already developed, stayed. Any excessive or abnormal flux should of course be met by every means in our power, but these means are often utterly inefficient unless accompanied by treatment directed to the uterus itself; thus diabetes and hæmaturia, leucorrhœa and hæmorrhoids, chronic dysentery, salivation and galactorrhœa, may all, while explaining the existence of amenorrhœa, themselves be kept up by the very quiescence of the uterus; so that, paradoxical as it may seem, to remove the cause it is here also necessary to remove its effect.

To the classes I have now enumerated I must add one other, none the less interesting from its rarity; the instances, namely, where the ovaries are absent, either congenitally or by operation, though the uterus is present. In the latter instance, the case at once declares itself by its history; in the former it is generally only after a long study that the diagnosis can finally be reached. As to treatment, we can only content ourselves with that recommended for absence of the uterus, to combat any general or local plethora that may exist, and for the rest patiently to withhold the hand.

